# Editorial Comment to Prognostic implication of staging of seminal vesicle invasion in patients with prostatic adenocarcinoma after prostatectomy

**DOI:** 10.1111/iju.14660

**Published:** 2021-07-31

**Authors:** Shinya Yamamoto

**Affiliations:** ^1^ Department of Genitourinary Oncology Japanese Foundation for Cancer Research Cancer Institute Hospital Tokyo Japan

Although seminal vesicle invasion (SVI) has been well documented as a poor prognostic factor, prostate cancer (PCA) with SVI is not associated with a uniformly poor prognosis. Given that the 5‐year biochemical recurrence (BCR)‐free survival rate of patients with pathological SVI (pT3b) has ranged from 33.8% to 38%, approximately one‐third of these patients might be cured by surgery alone.^1^ In this context, the classification of pT3b patients is useful in clinical practice to predict the prognosis and select the adjuvant treatment. To date, some investigators have reported on the volume and extent of the tumor in the seminal vesicle to classify the pT3b PCA.^2^, ^3^ The findings of these reports have been controversial and inconclusive. Debras *et al*. classified 52 patients with pT3b PCA into two groups by the extent of SVI. This grouping, along with the Gleason score, was a predictor of BCR in the cohort.^2^ Epstein *et al*. reported that the SVI extent was not a predictor of BCR in multivariate analysis.^3^ Yamamoto *et al*. measured the cancer volume involving the seminal vesicles (CVSVs) using a grid method. In multivariate analysis, both PSA level and CVSVs were identified as significant and independent predictors of BCR.^1^ Although CVSVs is a strong prognostic predictor for pT3b patients, CVSVs measurement can be complex.

In this study, Fukunaga *et al*. investigated the extent of SVI on the pathological specimen as a prognostic predictor.^4^ They classified SVI into three groups according to the maximum longitudinal tumor spread. Level 1 was defined as invasion in the proximal lesion of the seminal vesicles, level 2 was defined as invasion at the parallel line in the back of the prostate and level 3 was defined as invasion in the remaining distal lesion of the seminal vesicles. The BCR‐free survival rates of the three levels were significantly different (*P* = 0.002), and the extent of SVI was an independent predictor of BCR in multivariate analysis. This is a simple evaluation method that can be carried out by a pathologist during their routine microscopic examination.

There were a few limitations in this study. First, the sample size was too small. Furthermore, because most clinical T3b PCA patients received neoadjuvant hormonal therapy, they were excluded from this study cohort. Accordingly, only some of the pT3b PCA cases, which might be relatively early pT3b, were included in this cohort. Despite these limitations, this study is valuable and useful for clinical practice. In the future, I would like to see analysis using this classification carried out on more advanced pT3b PCA patients. Furthermore, it will be interesting to determine if this classification is associated with cancer‐specific survival and metastasis‐free survival.

## Conflict of interest

None declared.
